# Urbanity as a determinant of exposure to grass pollen in Helsinki Metropolitan area, Finland

**DOI:** 10.1371/journal.pone.0186348

**Published:** 2017-10-12

**Authors:** Timo T. Hugg, Jan Hjort, Harri Antikainen, Jarmo Rusanen, Mirkka Tuokila, Sanna Korkonen, Jan Weckström, Maritta S. Jaakkola, Jouni J. K. Jaakkola

**Affiliations:** 1 Center for Environmental and Respiratory Health Research, University of Oulu, Oulu, Finland; 2 Medical Research Center Oulu, University of Oulu, Oulu University Hospital and University of Oulu, Oulu, Finland; 3 Geography Research Unit, University of Oulu, Oulu, Finland; 4 Department of Environmental Sciences, University of Helsinki, Helsinki, Finland; University of Texas Health Science Center at San Antonio, UNITED STATES

## Abstract

Little is known about the levels of exposure to grass pollen in urban environments. We assessed the spatio-temporal variation of grass pollen concentrations and the role of urbanity as a determinant of grass pollen exposure in the Helsinki Metropolitan area. We monitored grass pollen concentrations in 2013 at 16 sites during the peak pollen season by using rotorod-type samplers at the breathing height. The sites were in the cities of Helsinki and Espoo, Finland, and formed city-specific lines that represented urban-rural gradient. The monitoring sites were both visually and based on land use data ranked as high to low (graded 1 to 8) pollen area. The lowest grass pollen concentrations were observed in the most urban sites compared to the least urban sites (mean 3.6 vs. 6.8 grains/m^3^ in Helsinki; P<0.0001, and 5.2 vs. 87.5 grains/m^3^ in Espoo; P<0.0001). Significant differences were observed between concentrations measured in morning periods compared to afternoon periods (4.9 vs. 5.4 in Helsinki, P = 0.0186, and 21.8 vs. 67.1 in Espoo, P = 0.0004). The mean pollen concentration increased with decreasing urbanity both in Helsinki (0.59 grains/m^3^ per urbanity rank, 95% CI 0.25–0.93) and Espoo (8.42, 6.23–10.61). Pollen concentrations were highest in the afternoons and they were related to the ambient temperature. Urbanity was a strong and significant determinant of pollen exposure in two Finnish cities. Pollen exposure can periodically reach such high levels even in the most urban environments that can cause allergic reactions among individuals with allergies.

## Introduction

Approximately 500 million people worldwide have been estimated to suffer from allergic rhinitis, and more than 300 million people from asthma [1–3]. The symptoms experienced by the majority of patients with asthma and/or allergic rhinitis increase during the pollen season [4, 5]. The treatment of the symptoms and signs include avoidance of pollen exposure and prescription of asthma and allergy medications. Most physicians are well informed of the current state of the art medicine treatment, whereas advising about avoidance of pollen exposure may be challenging because of lack of knowledge on the distribution and abundance of pollen during the pollen season. Majority of the current knowledge on pollen exposure levels is based on measurements at monitoring stations located on roofs, i.e. 10–20 meters above the ground level [6–8]. These monitoring stations are likely to give a good idea about the variation of pollen levels over time, but they do not provide information on spatial variation at the breathing height, which would be the most relevant information for asthma and allergy patients.

Grasses (*Poaceae*), including more than 10,000 species, grow in every continent and in almost all types of environments. Grasslands have been estimated to constitute 20–30% of the vegetation covering the earth [9]. Global distribution and abundance of grasses makes it difficult to totally avoid pollen exposure. Thus, grass pollen constitutes the most widespread group of pollen allergens worldwide. Grass is the most frequent cause of pollen allergy in Europe, and it is also among the most frequent allergens in the U.S [10, 11].

The growth conditions in urban environments differ much from those in rural environments. Differences in the urban vs. rural environments include factors such as discrete man-made/man-modified plant communities, limited amount of urban habitats, frequent disturbance or management by people, efficient construction/land use, higher urban temperatures, increased turbulences, lower levels of relative humidity, increased precipitation, and altered soil and air quality circumstances [12]. The specific environmental conditions in urban areas combined with the anthropogenic rise of atmospheric CO_2_ will affect the reproductive efficiency of plants, spreading of pollen, distribution and abundance of urban grasses, and finally, people’s exposure to allergens.

In general, average daytime CO_2_ concentrations as well as maximum daytime and minimum nighttime daily temperatures have increased in the urban compared to rural environments [13]. Elevated levels of CO_2_ have been shown to increase the grass pollen production by 50% per flower [14]. Additionally, allergenic potential of pollen in polluted urban areas has been suggested to be stronger than that of pollen grains in non-polluted rural areas [15, 16]. Urbanization in combination with potentially more favorable growth conditions leading to higher pollen counts and exposure, and greater pollen potency, may lead to markedly increased prevalence and severity of allergic symptoms and/or incidence of allergic diseases [17–21]. Haahtela and colleagues [22] have suggested as an extension of hygiene hypothesis [23] denoted as biodiversity hypothesis that a loss in biodiversity in urban environments may play a role in the etiology of allergies. There is some evidence that loss of the macrodiversity (reduced biodiversity) which characterizes urban environments is associated with shrinking of the microdiversity. Microdiversity loss is associated with alterations of the indigenous microbiota. These altered indigenous microbiota and the general microbial deprivation characterizing the environments and lifestyle of urban people in affluent countries appear to be risk factors for immune dysregulation, impaired tolerance and consequently allergic diseases [22, 24].

We recently monitored grass pollen levels in 16 sites placed at the breathing height in the Helsinki Metropolitan area during the peak pollen season. The location of sites and timing of measurements were designed to capture urban-rural variation in this area. We have already shown that land-use regression can be used to explain 79% of the spatial variation in the grass pollen data; a remote sensing based vegetation index was the strongest predictor of pollen concentrations [25]. In the majority of urban pollen studies, data are based on one or few monitoring stations [26, 27]. Only a few studies have actually presented measurements of pollen levels in several (more than three) urban environments and none of them have studied urbanization as a determinant of pollen exposures [28, 29]. The objective of the current article was to present the spatio-temporal variation of grass pollen concentrations during the peak season based on the above-mentioned 16 sites over 28 days. The second objective was to assess the role of urbanity as a determinant of grass pollen exposure at the breathing height. We also evaluated our measurements in different sites with respect to suggested clinically meaningful threshold levels, i.e. from 10 to 60 grains per m^3^ observed in previous studies and Finnish national threshold values [28, 30–32]. To our knowledge, this is the first study to extensively elaborate potential relations between different levels of urbanization and grass pollen concentrations at the breathing height in the context of changing climate. This is also the first study to elaborate diurnal variation of pollen concentrations in urban environments.

## Materials and methods

### Study area

**Fig 1:**
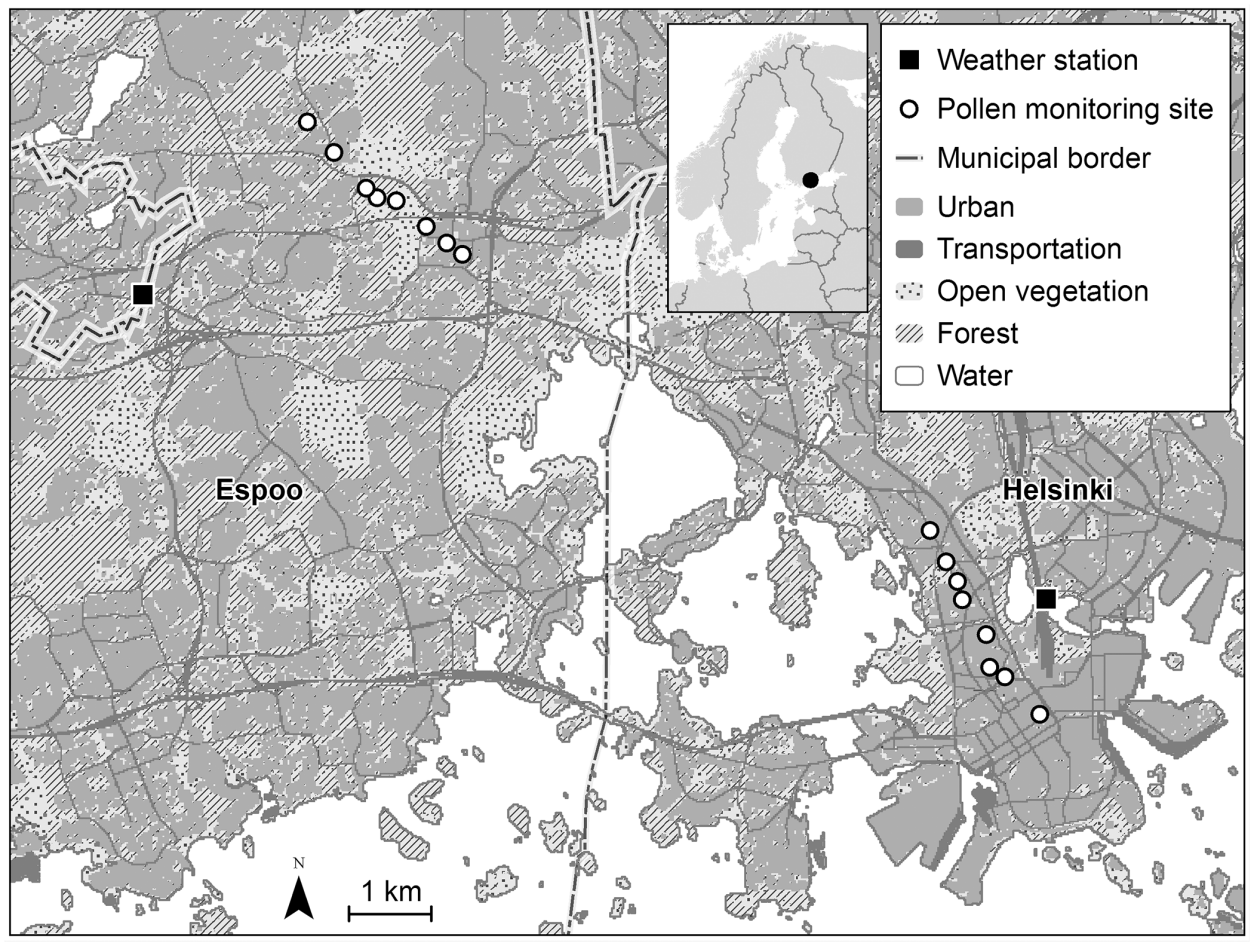
Map of the study area. Background map contains data from the General map 1:4 500 000 by the National Land Survey of Finland and the CLC2012 dataset by SYKE (partly Metla, Mavi, LIVI, VRK, MML Maastotietokanta 05/2012), licensed under a Creative Commons BY 4.0 International License (http://creativecommons.org/licenses/by/4.0/).

The study was conducted in the neighboring cities of Helsinki (60°10′15″N, 024°56′15″E) and Espoo (60°12′20″N, 024°39′20″E) in Southern Finland (Fig 1). Helsinki is the capital and the largest city of Finland, with 622 000 inhabitants, and Espoo is the second largest city in Finland, with 266 000 inhabitants. Helsinki, Espoo and the surrounding cities comprise the Helsinki Metropolitan area with altogether 1.1 million inhabitants. The population density is 2.930 and 858 inhabitants per km^2^ in Helsinki and in Espoo, respectively (*Statistics*; www.HelsinkiRegion.fi). The study area is located in Southern Finland between the Baltic Sea and the Eurasian continent, and thus, has characteristics of both maritime and continental climates. The mean annual temperature and the mean monthly temperature in July are +5.9°C and +17.8°C, respectively. The mean annual precipitation and the mean monthly precipitation in July are 655 mm and 63 mm, respectively, in these study areas (*Finnish Meteorological Institute*; http://ilmatieteenlaitos.fi/). Grasses constitute a typical pioneer plant group in the ground layer of the area. The study areas typically include both the effectively constructed urban environments with limited amount of vegetation and more or less natural environments covered by diverse vegetation

### Assessment of urbanity

The determinant of interest was urbanity. Urbanity of each sampling site was evaluated using two approaches. First, a city-specific ranking from 1 (most urban) to 8 (least urban) was conducted visually applying the following criteria: construction efficiency (scoring 1–10), street network coverage (1–10), and quantity of vegetation (10–1). From zero to ten points were given in each category (1 = natural environment and/or extremely lightly modified environment, 10 = man-made and/or extremely strongly modified environment. As an aberration, quantity of vegetation where 1 refers to man-made and 10 refers to natural environments). The division of each category followed so called 10% rule. Each change in urbanity score corresponded about 10% change in construction efficiency, street network coverage and quantity of vegetation. The scoring of each sampling site was based on total points given by the two researchers as a compromise of their opinion. Secondly, urbanity was assessed by applying Separated Land Use and Land Cover Information System [33] (Tables 1–3). The number of inhabitants [34] as well as the surface area of urban land use were estimated in the sampling sites. Because of limited dispersal capacity of grass pollen [35], urban land use and population data within a 100 meter radius from the sampling sites were used in the analyses.

**Table 1:** Environmental determinants of grass pollen concentration used in the analyses.

Determinant	Unit	Description	Source
**URB_GRAD**	Numeric value (1–8)	Urban gradient (urbanity rank; scoring 1 to 10)	Visual classification
**AM_PM**	Numeric value (1 or 2)	Measurement period; AM = morning (8:00–11:30); PM = afternoon (13:00–16:30)	Rotorod -type measurements
**POPUL100**	Numeric value (0–888)	Number of inhabitants within a 100 meter radius of the sampling sites	SeutuCD'12^a^
**URB_B100**	m^2^	Surface area of urban land use within a 100 meter radius of the sampling sites	SLICES land use classification^b^
**TEMP_PER**	°C	Mean air temperature within sampling period	Finnish Meteorological Institute
**DEWP_PER**	°C	Mean dew point within sampling period	Finnish Meteorological Institute
**HUM_PER**	%	Mean relative humidity within sampling period	Finnish Meteorological Institute
**WIN_PER**	m/s	Mean wind velocity within sampling period	Finnish Meteorological Institute
**GUS_PER**	m/s	Mean gust velocity within sampling period	Finnish Meteorological Institute
**PRE_PER**	hPa	Mean air pressure within sampling period	Finnish Meteorological Institute
**CLO_PER**	Numeric value (0–8)	Mean cloudiness within sampling period	Finnish Meteorological Institute

^a^SeutuCD'12. Helsinki Region Environmental Services Authority (HSY), Helsinki, 2012.

^b^SLICES data were from 2012 [National Land Survey of Finland (http://www.maanmittauslaitos.fi/en/kartat)].

**Table 2:** Characteristics of the study sites along the urban-rural gradient in the cities of Helsinki and Espoo.

Study site	Construction Efficiency^c^	Street Network Coverage^d^	Quantity of Vegetation^e^	Population Density (no per 100m radius)^f^	Urban Land Use (area per 100m radius)^g^
**Helsinki 1**^a^	9	7	1	814	30 100
**Helsinki 2**	9	7	2	888	31 100
**Helsinki 3**	8	5	2	16	28 700
**Helsinki 4**	6	8	3	133	31 600
**Helsinki 5**	4	4	7	205	21 100
**Helsinki 6**	3	4	7	269	24 800
**Helsinki 7**	4	4	8	602	24 000
**Helsinki 8**^b^	4	4	8	215	21 800
**Espoo 1**^a^	7	5	4	697	21 200
**Espoo 2**	7	4	4	276	23 500
**Espoo 3**	6	4	5	641	31 400
**Espoo 4**	5	3	5	335	28 800
**Espoo 5**	3	3	7	53	14 500
**Espoo 6**	3	2	8	3	5 300
**Espoo 7**	2	2	8	4	7 500
**Espoo 8**^b^	1	1	9	0	3 700

^a^Most urban.

^b^Least urban.

^c^Points of construction efficiency (1–10), where 1 represents natural environment and/or extremely lightly modified environment and correspondingly 10 represents man-made and/or extremely strongly modified environment.

^d^Street network coverage (1–10), where 0 represents natural environment and/or extremely lightly modified environment and correspondingly 10 represents man-made and/or extremely strongly modified environment.

^e^Quantity of vegetation (10–1), where 10 represents natural environment and/or extremely lightly modified environment and correspondingly 1 represents man-made and/or extremely strongly modified environment.

^f^Number of inhabitants within a 100 meter radius of the study site.

^g^Surface area of urban land use in square meters within a 100 meter radius of the study site. The highest values reflect the highest level of urbanization.

**Table 3:** Mean, median, percentile, minimum and maximum values of grass pollen concentrations (pollen grains/m3) at the study sites in the cities of Helsinki and Espoo.

Morning (n = 19–24)				Afternoon (n = 13–20)				
Study site	Mean	Median	Percentiles^c^	Min-Max	Mean	Median	Percentiles^c^	Min-Max
**Helsinki 1**^a^	2.55	1.5	0.0; 3.0	0–17	4.38	3.0	0.0; 7.5	0–23
**Helsinki 2**	2.96	3.0	0.0; 3.0	0–11	3.50	1.5	0.0; 3.0	0–20
**Helsinki 3**	1.29	0.0	0.0; 3.0	0–9	2.94	3.0	0.0; 4.5	0–11
**Helsinki 4**	10.33	3.0	0.0; 7.5	0–86	2.25	3.0	0.0; 3.0	0–6
**Helsinki 5**	4.71	3.0	0.0; 6.0	0–23	9.13	3.0	0.0; 10.0	0–54
**Helsinki 6**	2.76	3.0	0.0; 3.0	0–9	4.47	6.0	0.0; 6.0	0–14
**Helsinki 7**	5.90	3.0	0.0; 7.5	0–31	8.69	6.0	3.0; 10.0	0–37
**Helsinki 8**^b^	15.79	3.0	0.0; 11.0	0–119	9.50	6.0	3.0; 10.0	0–45
**Espoo 1**^a^	3.59	1.5	0.0; 6.0	0–20	5.40	6.0	1.5; 9.0	0–11
**Espoo 2**	8.08	6.0	1.5; 11.0	0–45	2.44	3.0	0.0; 3.0	0–14
**Espoo 3**	5.14	3.0	0.0; 6.0	0–20	8.26	6.0	3.0; 9.0	0–28
**Espoo 4**	3.04	3.0	0.0; 4.5	0–14	7.20	3.0	0.0; 10.0	0–34
**Espoo 5**	2.90	0.0	0.0; 0.3	0–20	14.67	6.0	3.0; 14.0	0–85
**Espoo 6**	8.24	6.0	0.0; 9.0	0–57	9.75	9.0	4.5; 12.5	3–28
**Espoo 7**	11.45	6.0	1.5; 9.0	0–57	81.85	9.0	0.0; 26.0	0–648
**Espoo 8**^b^	168.84	34.0	9.0; 151.0	0–1 545	549.79	54.0	11.0; 185.0	0–5 024

^a^Most urban.

^b^Least urban.

^c^The 25th and the 75th percentiles. A percentile is a measure used in statistics indicating the value below which a given percentage of observations in a group of observations fall. The 25th and 75th percentiles are the values below which 25% and 75% of the observations may be found.

### Assessment of grass pollen levels

Grass pollen concentration (exposure) at the breathing height, i.e. pollen grains per cubic meter of air sampled, was the outcome of interest. Pollen sampling was conducted at two lines of three kilometers that captured the urban-rural gradient within the cities of Helsinki and Espoo (Fig 1). In total, pollen grains were monitored at 16 points using rotorod-type samplers [36]. Samplers were equipped with a U-shaped metal rod (1.7 mm in diameter) and a power source (battery: NX, Powerfit S312/1.2S, Part No: AMP9033). Battery was fully charged at the start of each day. Transparent Melinex tape coated with an adhesive (Vaseline) was fixed to the upper ends of the rods. The speed of rotation of each sampler was monitored with a Shimpo Instruments (Itasca, Illinois, US) Shimpo DT-201 digital tachometer to ensure correct performance. The average speed of rotation of the arms was 2,173 rpm, varying between 2,040 and 2,275 rpm.

Samplers were attached to the top of the sampling poles at the height of 1.5 meters corresponding the average breathing height of humans. The sampling was conducted on days with no rainfall during the peak grass pollen season, i.e. between 27 June and 21 July 2013. The sampling period was 30 minutes. Sampling was conducted in each site twice a day: in the morning between 8.00 and 11.30 am and in the afternoon between 1 pm and 4.30 pm. Two researchers moved simultaneously in the line being in charge of either urban or rural pairs so that one started monitoring from the first sampling site (sites 1–4) and the other from the last sampling site (sites 8–5). The sampling progressed in time and was conducted within lines as a cycle of four days. The first sampling site (sites 3 and 6; sampling was conducted in the morning between 8:00 and 8:30 and in the afternoon between 13:00 and 13:30) in the first day was the fourth site (11:00 and 11:30; 16:00 and 16:30) in the second day, the third (10:00 and 10:30; 15:00 and 15:30) in the third day and the second (9:00 and 9:30; 14:00 and 14:30) in the fourth day. On the fifth day, rotation started from the beginning so that sampling was conducted in the morning between 8:00 and 8:30 and in the afternoon between 13:00 and 13:30 in these sampling sites. Other sampling sites followed the same rotation pattern in relation to time. The daily rotation occurred within morning and afternoon hours. Order of progress was identical in the morning and afternoon sessions. The protocol was designed to provide a balanced assessment of spatio-temporal variation in pollen concentration.

Pollen concentrations were determined by optical microscopic (Olympus BX43, Olympus Corporation, Tokyo, Japan) counting from collection tapes with 400 x magnification. A single transect line in the middle of the sample was examined; this comprised 32% of the total sample [37–39]. Pollen measurements were converted into volumetric equivalents, expressed as the amount of pollen grains per cubic meter of air sampled (grains/m^3^) [40].

### Covariates

Measurement period (i.e. morning or afternoon) and temperature were used as covariates in the multivariate analyses. Weather-related data were obtained from the Finnish Meteorological Institute. The two background weather stations were located in Kaisaniemi, Helsinki, and in Sepänkylä, Espoo. The distance from the background weather stations to the sampling sites varied from 1.1 to 2.3 km in Helsinki and from 2.9 to 3.8 km in Espoo (Fig 1). The following meteorological parameters during the sampling periods were used in the analyses: air temperature (°C), dew point (°C), relative humidity (%), wind velocity (m/s), gust (m/s), sea-level pressure (hPa) and cloudiness. Cloudiness was classified from zero (cloudless) to eight (totally cloudy; see Table 1).

### Statistical methods

The graphical illustration of data and the Kolmogorov-Smirnov Goodness-of-Fit test indicated that the data were not normally distributed. Therefore, we applied non-parametric statistical methods. We used the Wilcoxon two-sample test for paired comparison of daily pollen concentrations between more and less urban sampling sites. The Spearman correlation coefficient was used to evaluate potential associations between environmental variables and pollen concentrations, as well as potential associations between different environmental variables. Because of the zero-inflated distribution of pollen data, negative binomial regression modelling (applying proc GENMOD procedure) was used to assess the relation between environmental factors (independent variables) and pollen concentration (dependent variable) along the urban-rural gradient. To investigate the effect of daily variation of temperature on pollen concentration, temperature was analyzed by quartiles. Only temperature was selected to be included in the final model from the several inter-correlated weather-related factors, because it was most strongly associated with pollen concentrations. The results of two rods were dealt with as a two separate measurements. Analyses were performed applying the SAS software (*SAS 9*.*4*, *SAS Institute*, *Inc*., *Cary*, *NC*, *U*.*S*.). Arithmetic means, medians, 25^th^ and 75^th^ percentiles, and minimum and maximum values of the grass pollen are presented.

## Results

The construction efficiency and street network coverage decreased, while the quantity of vegetation increased when moving from more urban to less urban sampling sites in both cities (Table 2). The number of inhabitants and the surface area covered by urban land use in square meters (within a 100 meter radius of the sampling site) were consistent with the visual assessment of urban-rural pattern, although there were some deviations noticed from this pattern.

In general, pollen concentrations varied moderately within sampling sites and between monitoring periods. Zero concentrations were relatively common in all sampling sites in both cities, during both morning and afternoon periods (Table 3). However, pollen concentrations reached periodically the level from moderate (10–30) to abundant (>30 grains/m^3^) even in the most urban sampling sites. To our surprise, concentrations did not increase monotonously when moving from more to less urban sampling sites. The Finnish national threshold value for abundant grass pollen (i.e. >30 grains/m^3^ [32]) was exceeded twice during the sampling period in the four most urban environments, both in Helsinki and Espoo. Pollen concentrations were above the threshold value 6 and 28 times during the sampling period in the four least urban environments in Helsinki and Espoo, respectively.

The mean grass pollen concentrations were lower in the four most urban sampling sites compared to such means in the four least urban sampling sites (overall mean 3.6 vs. 6.8 grains/m^3^ in Helsinki; P<0.0001, and 5.2 vs. 87.5 grains/m^3^ in Espoo; P<0.0001). In addition, the mean grass pollen concentrations were lower during the morning periods compared to the afternoon periods (overall mean 4.9 vs. 5.4 grains/m^3^ in Helsinki; P = 0.0186, and 21.8 vs. 67.1 grains/m^3^ in Espoo; P = 0.0004). Pollen concentrations correlated significantly with urban gradient (correlation coefficient (r) -0.236; P<0.0001), urban land use (-0.179; P = 0.0018), and air pressure (0.114; P = 0.0471) in Helsinki (Table 4). Correspondingly, pollen concentrations correlated significantly with the air temperature (0.337; P<0.0001), the urban gradient (-0.318; P<0.0001), the number of inhabitants (-0.307; P<0.0001), urban land use (-0.285; P<0.0001), humidity (-0.195; P = 0.0006), air pressure (0.186; P = 0.0011), and sampling period (0.155; P = 0.0066) in Espoo (Table 5).

**Table 4:** Spearman’s correlations coefficients (r_s_) and p-values for the relationship between the grass pollen concentrations and environmental variables in Helsinki (n = 368).

**Variable**	**POLLEN**^a^	**URB_GRAD**	**AM_PM**	**POPUL100**	**URB_B100**	**TEMP_PER**	**DEWP_PER**	**HUM_PER**	**WIN_PER**	**GUS_PER**	**PRE_PER**	**CLO_PER**^b^
**POLLEN**^a^	1.000	-0.236*	0.089	0.048	-0.179*	0.038	0.099	0.058	0.019	0.032	0.114*	0.046
**URB_GRAD**		1.000	0.000	-0.185*	-0.694*	0.009	0.023	0.021	0.003	0.005	0.007	0.021
**AM_PM**			1.000	0.000	0.000	0.313*	0.103*	-0.136*	0.285*	0.269*	0.015	0.054
**POPUL100**				1.000	0.111*	-0.016	-0.033	-0.047	0.041	0.002	-0.007	-0.056
**URB_B100**					1.000	0.011	0.007	-0.000	-0.001	0.002	0.004	0.001
**TEMP_PER**						1.000	0.252*	-0.381*	-0.249*	-0.367*	0.350*	-0.417*
**DEWP_PER**							1.000	0.760*	-0.339*	-0.263*	0.375*	0.331*
**HUM_PER**								1.000	-0.246*	-0.121*	0.231*	0.553*
**WIN_PER**									1.000	0.913*	-0.430*	-0.038
**GUS_PER**										1.000	-0.491*	0.112*
**PRE_PER**											1.000	0.002
**CLO_PER**^b^												1.000

Abbreviations: POLLEN, pollen concentration (pollen grains per m3 of air); URB_GRAD, urban scoring of sampling sites (sites 1–8); AM_PM, morning vs. afternoon; POPUL100, Number of inhabitants within a 100 meter radius of the study site; URB_B100, Surface area of urban land use in square meters within a 100 meter radius of the study site; TEMP_PER, mean air temperature within sampling period; DEWP_PER, mean dew point within sampling period; HUM_PER, mean relative humidity within sampling period; WIN_PER, mean wind velocity within sampling period; GUS_PER, mean gust velocity within sampling period; PRE_PER, mean air pressure within sampling period; CLO_PER, mean cloudiness within sampling period.

^a^n = 302.

^b^n = 352.

* P < 0.05

**Table 5:** Spearman’s correlations coefficients (r_s_) and p-values for the relationship between the grass pollen concentrations and environmental variables in Espoo (n = 384).

**Variable**	**POLLEN**^a^	**URB_GRAD**	**AM_PM**	**POPUL100**	**URB_B100**	**TEMP_PER**	**DEWP_PER**	**HUM_PER**	**WIN_PER**	**GUS_PER**	**PRE_PER**	**CLO_PER**^b^
**POLLEN**^a^	1.000	-0.318*	0.155*	-0.307*	-0.285*	0.337*	0.032	-0.195*	-0.058	-0.083	0.186*	.
**URB_GRAD**		1.000	0.000	-0.905*	-0.762*	0.000	0.000	0.000	0.000	0.000	0.000	.
**AM_PM**			1.000	0.000	0.000	0.532*	-0.131*	-0.447*	0.416*	0.434*	-0.068	.
**POPUL100**				1.000	0.857*	0.003	-0.013	-0.011	0.021	0.016	0.001	.
**URB_B100**					1.000	0.014	-0.030	-0.032	0.021	0.019	-0.001	.
**TEMP_PER**						1.000	-0.148*	-0.700*	-0.017	-0.012	0.325*	.
**DEWP_PER**							1.000	0.774*	-0.116*	-0.131*	0.005	.
**HUM_PER**								1.000	-0.064	-0.082	-0.177*	.
**WIN_PER**									1.000	0.968*	-0.362*	.
**GUS_PER**										1.000	-0.350*	.
**PRE_PER**											1.000	.
**CLO_PER**^b^												.

Abbreviations: POLLEN, pollen concentration (pollen grains per m^3^ of air); URB_GRAD, urban scoring of sampling sites (sites 1–8); AM_PM, morning vs. afternoon; POPUL100 = Number of inhabitants within a 100 meter radius of the study site; URB_B100, Surface area of urban land use in square meters within a 100 meter radius of the study site; TEMP_PER, mean air temperature within sampling period; DEWP_PER, mean dew point within sampling period; HUM_PER, mean relative humidity within sampling period; WIN_PER, mean wind velocity within sampling period; GUS_PER, mean gust velocity within sampling period; PRE_PER, mean air pressure within sampling period; CLO_PER, mean cloudiness within sampling period.

^a^n = 306.

^b^n = . (no observations available).

* P < 0.05

In the negative binomial regression analysis adjusting for ambient temperature and the time of the day, the mean pollen concentration increased with decreasing urbanity rank in Helsinki (0.59 grains/m^3^ per urbanity rank, 95% CI: 0.25–0.93). Correspondingly, the mean pollen concentration increased with decreasing urbanity rank (8.42, 6.23–10.61) and decreasing efficiency of urban land use (1.90, 1.33–2.48) in Espoo. In addition, differences in air temperature explained largely the variation observed in pollen concentrations between the sampling sites (Table 6). Pollen concentration in Helsinki increased by 0.79–0.83 grains/m^3^ (Estimates: 0.79, 95% CI 0.35–1.22; and 0.83, 95% CI 0.41–1.24) per one degree Celsius increase during the sampling period, and by 0.48 grains/m^3^ (0.48, 0.32–0.63) per one degree Celsius in Espoo. Interestingly, highest increases in the mean pollen concentrations were not observed during the hottest period of day (4^th^ quartile) but during the quartile before it (3^rd^ quartile; Table 6).

**Table 6:** The relationship between the grass pollen concentration and environmental factors in the negative binomial regression model (Helsinki n = 302, Espoo n = 306).

	POLLEN Helsinki (pollen grains / m^3^)		POLLEN Espoo (pollen grains / m^3^)	
Predictors	Estimate (95% CI)	P value	Estimate (95% CI)	P value
**Model 1**				
Urban gradient (1 to 8)	**0.5872** (0.2488, 0.9256)	0.0007	**8.4192** (6.2258, 10.6126)	<.0001
Sampling period (AM = 1, PM = 2)	-0.8690 (-2.9056, 1.1676)	0.4030	0.0535 (-5.3589, 5.4659)	0.9845
Temperature (per 1°C)	**0.7857** (0.3537, 1.2177)	0.0004	0.5991 (-0.4076, 1.6057)	0.2435
**Model 2**				
Urban land use (m^2^)^a^	0.3027 (-0.1019, 0.7074)	0.1426	**1.9042** (1.3248, 2.4836)	<.0001
Sampling period (AM = 1, PM = 2)	-1.4299 (-3.6616, 0.8019)	0.2092	3.4544 (-0.3716, 7.2804)	0.0768
Temperature (per 1°C)	**0.8287** (0.4142, 1.2432)	<.0001	**0.4758** (0.3242, 0.6273)	<.0001
**Model 3**				
Urban gradient (1 to 8)	**0.5211** (0.2092, 0.8330)	0.0011	**8.4121** (6.1438, 10.6804)	<.0001
Temperature 0 (1^st^ quartile; reference)^b^	0	0	0	0
Temperature 1 (2^nd^ quartile)^c^	0.7473 (-0.7627, 2.2572)	0.3321	0.0847 (-3.4442, 3.6135)	0.9625
Temperature 2 (3^rd^ quartile)^d^	**6.9816** (3.0396, 10.9236)	0.0005	2.2666 (-4.0882, 8.6215)	0.4845
Temperature 3 (4^th^ quartile)^e^	0.9770 (-0.8105, 2.7645)	0.2840	1.5113 (-2.6767, 5.6994)	0.4794

^a^Surface area of urban land use in square meters within a 100 meter radius of the study site.

^b^1^st^ quartile: + 14.40 –+ 17.85°C (Helsinki) and + 13.10 –+ 17.90°C (Espoo).

^c^2^nd^ quartile: + 17.86 –+ 19.10°C (Helsinki) and + 17.91 –+ 20.25°C (Espoo).

^d^3^rd^ quartile: + 19.11 –+ 20.60°C (Helsinki) and + 20.26 –+ 22.10°C (Espoo).

^e^4^th^ quartile: + 20.61 –+ 24.20°C (Helsinki) and + 22.11 –+ 25.80°C (Espoo).

## Discussion

### Main findings

To our knowledge, this is the first study that assessed systematically potential fine-scale variation in the exposure to grass pollen within different urban environments. The level of urbanization explained well the observed differences in the pollen exposure along the urban-rural gradient. In general, grass pollen exposure decreased with increasing level of urbanization in the Helsinki Metropolitan area. However, the decline in pollen exposure did not always follow a consistent pattern with increasing urbanity, although the trend was statistically significant. Thus, pollen concentrations in the 30-minute sampling periods occasionally reached high levels (i.e. >30 grains/m^3^ [32]) also in the most urban site. At such high levels individuals with asthma and allergies are likely to experience symptoms. From the allergological point of view, the most urban environments with small, discrete and frequently managed patches of vegetation have little potential to produce considerable amounts of pollen grains and to expose people to pollen. Thus, staying in urban environments will reduce the overall exposure to grass pollen, although occasionally higher levels of exposure may be encountered. Epidemiologic studies are needed to evaluate whether staying in urban environments actually reduces allergic reactions. Visual urban-rural gradient assessment showed its functionality and feasibility for pollen exposure studies. Although visual evaluation of urban environments is more subjective, it can give more precise picture of the immediate surroundings of the sampling sites in patchy urban environments than the more coarse-grained evaluation approaches (such as land use data and aerial photograph interpretations). This approach provides a new practical tool to assess the variation in the concentrations of pollen and can be used to evaluate exposure to pollen relevant for health effects in urban environments.

### Validity of results

Pollen concentrations have been observed to vary both vertically and horizontally in the urban environments [29, 41]. Therefore, only one or even several monitoring sites at only the roof level may not reflect accurately the exposure pattern of pollen relevant for health effects. In this study, we selected a total of 16 sampling sites to capture a comprehensive spectrum of urban environments. In addition, this enabled us to measure more accurately the intra-urban spatial variation in pollen concentrations. We placed our samplers at a height of 1.5 meters, which enabled assessment of pollen concentrations at the breathing height, the most relevant measure of exposure for our health outcomes.

Due to a limited amount of resources, we were not able to organize the sampling at exactly the same point in time, but formed sampling site pairs (consisting of the most urban vs. least urban site etc.) where simultaneous sampling could be conducted. To control for potential temporal within-day variation in pollen concentrations, we conducted sampling twice a day (at 8.00–11.30 am and at 1.00–4.30 pm). In addition, the beginning of the sampling progressed by one hour per day both in the morning and afternoon sessions. Our sampling covered both the peak season and the daily peak flowering period of the grasses [42]. Helsinki and Espoo data were analyzed separately because their sampling had been conducted on alternate days. Each sampling period was restricted to 30 minutes to minimize potential problems due to oversampling where the tapes’ capacity to bind pollen was exceeded [43]. Although the 30-minute sampling period does not reflect the total diurnal pollen load, it gives an accurate estimate of the short-term pollen concentration (i.e. exposure), and its variation over time and space. This sampling period is also consistent with a rather short average duration of outdoor activities of dwellers in region [44].

The weather conditions may vary substantially even within short distances. Human activity in combination with man-made structures can strengthen the effects of weather, thus leading to pronounced variation in microclimatic weather conditions in urban environments [12]. Thus, results based on regional weather-related information from background weather stations should be considered suggestive and interpreted with caution. Our sampling was restricted to dry weather conditions, because pollen grains may be washed away with rain from the sampling surfaces and from the air [45].

### Synthesis with previous knowledge

To our knowledge, this is the first study to show fine-scale urban-rural gradient in pollen exposure at the breathing height based on volumetric sampling. Information on fine-scale exposure to allergenic pollen is critically important when spatial and temporal variation in pollen exposure and individual exposure to pollen are assessed and modelled [25]. Previous studies, conducted in France [28] and Spain [27] have provided some evidence of differences in pollen concentrations between urban and rural environments. Bosch-Cano et al. [28] showed in four geographically distinct sampling areas in France that the total allergenic pollen load was generally higher at the breathing height in non-urban compared to urban areas, consistently with the urban-rural gradient shown for grass pollen in the present study. A Spanish study conducted by Gonzalo-Garijo et al. [27] did not find any significant differences in grass pollen concentrations between three urban sampling sites located at ground level (10 cm). A recent study, conducted in Berlin, applied 14 gravimetric pollen traps placed at street-level height (2.0–3.5 m above ground). There were substantial spatial and temporal variations of grass pollen sedimentation within the city [46]. Most studies have monitored pollen concentrations on the roof level [29, 47] rather than at the breathing height and thus the pollen concentrations are not comparable with those of the present study.

In this study, pollen concentrations exceeded periodically moderate (10–30) and abundant (>30 grains/m^3^) values even in the most urban sampling sites [32]. Pollen peaks have been studied in a few previous studies at breathing height in urban environments. Consistently with our study, the grass pollen concentrations exceeded occasionally the clinically relevant threshold value (>20 grains/m^3^) in all three sampling sites located partly at the breathing height in the city of Berlin, Germany [48]. Correspondingly, a French study showed that the number of days above the allergenic threshold value (i.e. 10 grains/m^3^) for grasses were more common in the rural and semi-rural areas compared to the urban areas [28]. A study conducted in Islamabad, Pakistan used Rotorod samplers at roof level in five different sectors of the city between 2010 and 2012. The average daily grass pollen concentrations settled most commonly within the category of 0–25 pollen grains per m3 [49]. Because there is lack of comparable studies with assessment of clinical outcomes, we are not able to assess precisely the clinical effects related to such pollen exposure in urban environments.

Our results showed a substantial temporal variation in pollen concentrations both in the most urban and in rural sites which is consistent with results from urban environments in Denmark and UK [50]. There are two main reasons for the observed variation. First, vertical structures of the cities can efficiently prevent a free horizontal airflow of pollen grains producing gust and vortices. Therefore, gusts and vortices due to man-made vertical surfaces can drive the number of pollen grains from both immediate sources and sources located outside the city, promote re-takeoff of already settled pollen, and then expose people through an unpredictable way [12]. Second, pollen release height is generally rather low and the size of pollen grain is relatively large. Therefore, most of pollen settles in the immediate vicinity of grass growth, probably resulting in larger intra-urban (horizontal and vertical) variations in grass pollen concentrations compared to trees. This variation is mostly linked to local-scale variations in the distribution of grasses [47, 51].

The results of the present study underline the importance of weather-related factors for the occurrence of grass pollen in the urban environments. The effect of air temperature on pollen exposure was more pronounced in Helsinki than in Espoo, possibly reflecting more extensive heat release from man-made urban surfaces in Helsinki. This heat release can produce so called “heat island effect” where the most urban environments are warmed up the most [12], resulting in a small scale heat gradient between the sampling sites. Individuals are likely to be exposed to grass pollen during afternoon hours, often linked to increased windiness during the warm and dry time of the day, with typical conditions for high pressure in the region. Consistent with our study, grass pollen concentrations were partly associated with wind speed in the urban area of Poznan, Poland [8]. Correspondingly, relative humidity showed significant negative correlation with grass pollen concentrations, while hours of sunshine and daily maximum temperature showed positive correlations with grass pollen concentrations in Berlin, Germany [48, 52]. However, there can be small-scale variation between negative and positive correlations of grass pollen and humidity on hourly basis within day [53].

## Conclusions

Our results that are based on extensive pollen monitoring provide new evidence on fine-scale spatial and temporal variation in grass pollen exposure in urban environments. We show that urbanity level based on educated subjective assessment predicts average pollen exposure at the breathing height. By combining the predicted pollen levels with information (from health effects studies) on clinical effects related to such levels, this information could be used in clinical practice to advise allergic subjects to protect themselves from such exposures by avoiding risk areas and peak times, and thus, to prevent allergic reactions.

The local sources, such as unmanaged open lands, may substantially contribute to pollen exposure. Therefore, identification and avoidance the vicinity of such locations may reduce exposure and consequently, allergic reactions. The authorities should arrange their surveillance more extensively and transfer such information into the health care system, and for patients and patient organizations. Physicians and nurses should be educated about the determinants of the urban allergen load, so that they could advice their allergic patients to favor areas of lower pollen exposure (i.e. most urban parts of the city). Such advice could also include a recommendation to spend time outdoors outside the peak period of grass pollen (i.e. early in the morning). In this study, the peak concentrations were experienced during afternoons, which should be taken into account in timing and dosing of medications. It is worth noticing that high temperature increases pollen exposure levels during the pollen season.

Prevailing circumstances in the cities correspond to the near future conditions projected in the scenarios for climate change [13, 54]. Higher temperature and CO_2_ concentrations in the urban environments are likely to increase pollen production, and as a consequence, lead to higher exposure to pollen. Therefore, it is necessary to study and understand the determinants of exposure to pollen grains in the urban environments to facilitate effective preventive measures. The monitoring of pollen spatial dynamics enables identification of high exposure environments and thus, constitutes an important tool for the guidance of allergic patients by physicians and nurses.

## Supporting information

S1 Data The Helsinki grass pollen data 2013. (XLS)

## References

[1] Masoli M, Fabian D, Holt S, Beasley R. Global Initiative for Asthma (GINA) Program. The global burden of asthma: executive summary of the GINA Dissemination Committee report. Allergy. 2004; 59: 469–478. doi: 10.1111/j.1398-9995.2004.00526.x 15080825 10.1111/j.1398-9995.2004.00526.x

[2] Bousquet J, Khaltaev N, Cruz AA, Denburg J, Fokkens WJ, Togias A, et al Allergic Rhinitis and its Impact on Asthma (ARIA) 2008 update (in collaboration with the World Health Organization, GA(2)LEN and AllerGen). Allergy. 2008; 63 Suppl 86: 8–160. 18331513 10.1111/j.1398-9995.2007.01620.x

[3] Ozdoganoglu T, Songu M. The burden of allergic rhinitis and asthma. Ther Adv Respir Dis. 2012; 6: 11–23. doi: 10.1177/1753465811431975 22179899 10.1177/1753465811431975

[4] Blomme K, Tomassen P, Lapeere H, Huvenne W, Bonny M, Acke F, et al Prevalence of allergic sensitization versus allergic rhinitis symptoms in an unselected population. Int Arch Allergy Immunol. 2013; 160: 200–207. doi: 10.1159/000339853 23018768 10.1159/000339853

[5] Taylor PE, Jacobson KW, House JM, Glovsky MM. Links between pollen, atopy and the asthma epidemic. Int Arch Allergy Immunol. 2007; 144: 162–170. doi: 10.1159/000103230 17536216 10.1159/000103230

[6] Emberlin J, Norris-Hill J. Spatial variation of pollen deposition in north London. Grana. 1991; 30: 190–195.

[7] Arroba D, Guido MA, Minale P, Montanari C, Placereani S, Pracilio S, et al Airborne pollen in Genoa (NW-Italy): a comparison between two pollen-sampling stations. Aerobiologia. 2000; 16: 233–243.

[8] Rodríguez-Rajo FJ, Fdez-Sevilla D, Stach A, Jato V. Assessment between pollen seasons in areas with different urbanization level related to local vegetation sources and differences in allergen exposure. Aerobiologia. 2010; 26: 1–14.

[9] Constable G. Grasslands and Tundra Planet Earth Series. 1st ed Alexandria, VA: Time Life Books; 1985.

[10] D'Amato G, Cecchi L, Bonini S, Nunes C, Annesi-Maesano I, Behrendt H, et al Allergenic pollen and pollen allergy in Europe. Allergy. 2007; 62: 976–990. doi: 10.1111/j.1398-9995.2007.01393.x 17521313 10.1111/j.1398-9995.2007.01393.x

[11] White JF, Bernstein DI. Key pollen allergens in North America. Ann Allergy Asthma Immunol. 2003; 91: 425–435. doi: 10.1016/S1081-1206(10)61509-8 14692424 10.1016/S1081-1206(10)61509-8

[12] Douglas I. The Urban Environment. 1st ed London, UK: Edward Arnold Publishers Ltd; 1983.

[13] Ziska LH, Bunce JA, Goins EW. Characterization of an urban-rural CO2/temperature gradient and associated changes in initial plant productivity during secondary succession. Oecologia. 2004; 139: 454–458. doi: 10.1007/s00442-004-1526-2 15021982 10.1007/s00442-004-1526-2

[14] Albertine JM, Manning WJ, DaCosta M, Stinson KA, Muilenberg ML, Rogers CA. Projected carbon dioxide to increase grass pollen and allergen exposure despite higher ozone levels. PLoS One. 2014; 9(11): e111712 doi: 10.1371/journal.pone.0111712 25372614 10.1371/journal.pone.0111712 PMC4221106

[15] Aina R, Asero R, Ghiani A, Marconi G, Albertini E, Citterio S. Exposure to cadmium-contaminated soils increases allergenicity of *Poa annua L*. pollen. Allergy. 2010; 65: 1313–1321. doi: 10.1111/j.1398-9995.2010.02364.x 20374228 10.1111/j.1398-9995.2010.02364.x

[16] Majd A, Chehregani A, Moin M, Gholami M, Kohno S, Nabe T, et al The effects of air pollution on structures, proteins and alleregenicity of pollen grains. Aerobiologia. 2004; 20: 111–118.

[17] Armentia A, Banuelos C, Arranz ML, Del Villar V, Martin-Santos JM, Gil FJ, et al Early introduction of cereals into children’s diets as a risk-factor for grass pollen asthma. Clin.Exp.Allergy. 2001; 31: 1250–1255. 11529895 10.1046/j.1365-2222.2001.01142.x

[18] Priftis KN, Anthracopoulos MB, Nikolaou-Papanagiotou A, Matziou V, Paliatsos AG, Tzavelas G, et al Increased sensitization in urban vs. rural environment—rural protection or an urban living effect? Pediatr Allergy Immunol. 2007; 18: 209–216. doi: 10.1111/j.1399-3038.2006.00514.x 17432999 10.1111/j.1399-3038.2006.00514.x

[19] Beggs PJ. Impacts of climate change on aeroallergens: past and future. Clin Exp Allergy. 2004; 34: 1507–1513. doi: 10.1111/j.1365-2222.2004.02061.x 15479264 10.1111/j.1365-2222.2004.02061.x

[20] Gilmour MI, Jaakkola MS, London SJ, Nel AE, Rogers CA. How exposure to environmental tobacco smoke, outdoor air pollutants and increased pollen burdens influences the incidence of asthma. Environ Health Perspect. 2006; 114: 627–633. doi: 10.1289/ehp.8380 16581557 10.1289/ehp.8380 PMC1440792

[21] Ziello C, Sparks TH, Estrella N, Belmonte J, Bergmann KC, Bucher E, et al Changes to airborne pollen counts across Europe. PLoS One. 2012; 7: e34076 doi: 10.1371/journal.pone.0034076 22514618 10.1371/journal.pone.0034076 PMC3325983

[22] Haahtela T, Holgate S, Pawankar R, Akdis CA, Benjaponpitak S, Caraballo L, et al; WAO Special Committee on Climate Change and Biodiversity. The biodiversity hypothesis and allergic disease: world allergy organization position statement. World Allergy Organ J. 2013; 6(1): 3 doi: 10.1186/1939-4551-6-3 23663440 10.1186/1939-4551-6-3 PMC3646540

[23] Strachan DP. Hay fever, hygiene, and household size. BMJ. 1989; 299(6710): 1259–1260. 2513902 10.1136/bmj.299.6710.1259 PMC1838109

[24] Hanski I, von Hertzen L, Fyhrquist N, Koskinen K, Torppa K, Laatikainen T, et al Environmental biodiversity, human microbiota, and allergy are interrelated. Proc Natl Acad Sci U S A. 2012; 109(21): 8334–8339. doi: 10.1073/pnas.1205624109 22566627 10.1073/pnas.1205624109 PMC3361383

[25] Hjort J, Hugg TT, Antikainen H, Rusanen J, Sofiev M, Kukkonen J, et al Fine-scale exposure to allergenic pollen in the urban environment: evaluation of land use regression approach. Environ Health Perspect. 2016; 124: 619–626. doi: 10.1289/ehp.1509761 26452296 10.1289/ehp.1509761 PMC4858385

[26] Alcázar P, Galán C, Cariñanos P, Domínguez-Vilches E. Diurnal variation of airborne pollen at two different heights. J Investig Allergol Clin Immunol. 1999; 9(2): 85–89. 10353095

[27] Gonzalo-Garijo MA, Tormo-Molina R, Muñoz-Rodríguez AF, Silva-Palacios I. Differences in the spatial distribution of airborne pollen concentrations at different urban locations within a city. J Investig Allergol Clin Immunol. 2006; 16: 37–43. 16599247

[28] Bosch-Cano F, Bernard N, Sudre B, Gillet F, Thibaudon M, Richard H, et al Human exposure to allergenic pollens: A comparison between urban and rural areas. Environ Res. 2011; 111: 619–625. doi: 10.1016/j.envres.2011.04.001 21531404 10.1016/j.envres.2011.04.001

[29] Cariñanos P, Sánchez-Mesa JA, Prieto-Baena JC, Lopez A, Guerra F, Moreno C, et al Pollen allergy related to the area of residence in the city of Córdoba, south-west Spain. J Environ Monit. 2002; 4: 734–738. 12400923 10.1039/b205595c

[30] Annesi-Maesano I, Rouve S, Desqueyroux H, Jankovski R, Klossek JM, Thibaudon M, et al Grass pollen counts, air pollution levels and allergic rhinitis severity. Int Arch Allergy Immunol. 2012; 158: 397–404. doi: 10.1159/000332964 22487690 10.1159/000332964

[31] Feo Brito F, Mur Gimeno P, Carnés J, Fernández-Caldas E, Lara P, Alonso AM, et al Grass pollen, aeroallergens, and clinical symptoms in Ciudad Real, Spain. J Investig Allergol Clin Immunol. 2010; 20(4): 295–302. 20815307

[32] Ranta H, Pessi A-M (eds.). The Finnish Pollen Bulletin Summary. Aerobiology Unit, University of Turku, Finland; 2005.

[33] SLICES, 2012. SLICES land use. National Land Survey of Finland, Helsinki. http://www.maanmittauslaitos.fi/en/kartat.

[34] SeutuCD'12. Helsinki: Helsinki Region Environmental Services Authority (HSY); 2012.

[35] Raynor GS, Hayes JV, Ogden EC. Experimental data on dispersion and deposition of timothy and corn pollen from known sources. Upton, NY: Brookhaven National Laboratory; 1970.

[36] Raynor GS. Sampling particulates with rotating arm impaction samplers. In: Benninghoff WS, Edmonds RL, editors. Ecological system approaches to aerobiology. I Identification of component elements and their functional relationships. In: Proceedings of Workshop/Conference I, US/IBP Aerobiology Program Handbook Number 2. Ann Arbor: The University of Michigan; 1972. pp. 82–105.

[37] Galán C, Smith M, Thibaudon M, Frenguelli G, Oteros J, Gehrig R, et al Pollen monitoring: minimum requirements and reproducibility of analysis. Aerobiologia 2014; 30: 385–395.

[38] Rantio-Lehtimäki A, Helander ML, Karhu K. Does cutting of mugwort stands affect airborne pollen concentrations? Allergy. 1992; 47: 388–390. 1456409 10.1111/j.1398-9995.1992.tb02077.x

[39] Frenz DA, Scamehorn RT, Hokanson JM, Murray LW. A brief method for analyzing Rotorod samples for pollen content. Aerobiologia. 1996; 12: 51–54.

[40] Hugg T, Rantio-Lehtimaki A. Indoor and outdoor pollen concentrations in private and public spaces during the *Betula* pollen season. Aerobiologia. 2007; 23(2): 119–129.

[41] Rantio-Lehtimäki A, Koivikko A, Kupias R, Mäkinen Y, Pohjola A. Significance of sampling height of airborne particles for aerobiological information. Allergy. 1991; 46: 68–76. 2018211 10.1111/j.1398-9995.1991.tb00545.x

[42] Käpylä M. Diurnal Variation of Non-Arboreal Pollen in the Air in Finland. Grana. 1981; 20: 55–59.

[43] Sterling P, Lewis R. Pollen and fungal spores indoor and outdoor of mobile homes. Ann Allergy Asthma Immunol. 1998; 80: 279–285. doi: 10.1016/S1081-1206(10)62971-7 9532979 10.1016/S1081-1206(10)62971-7

[44] Jurvelin J, Vartiainen M, Jantunen M, Pasanen P. Personal Exposure Levels and Microenvironmental Concentrations of Formaldehyde and Acetaldehyde in the Helsinki Metropolitan Area, Finland. J Air & Waste Manage Assoc. 2001; 51: 17–24. 10.1080/10473289.2001.10464251 11218421

[45] Khwarahm N, Dash J, Atkinson PM, Newnham RM, Skjøth CA, Adams-Groom B, et al Exploring the spatio-temporal relationship between two key aeroallergens and meteorological variables in the United Kingdom. Int J Biometeorol. 2014; 58(4): 529–545. doi: 10.1007/s00484-013-0739-7 24482047 10.1007/s00484-013-0739-7

[46] Werchan B, Werchan M, Mücke HG, Gauger U, Simoleit A, Zuberbier T, et al Spatial distribution of allergenic pollen through a large metropolitan area. Environ Monit Assess. 2017; 189(4): 169 doi: 10.1007/s10661-017-5876-8 28316024 10.1007/s10661-017-5876-8

[47] Kasprzyk I. Comparative study of seasonal and intradiurnal variation of airborne herbaceous pollen in urban and rural areas. Aerobiologia. 2006; 22: 185–195.

[48] Bergmann KC, Simoleit A, Wagener S, Mücke HG, Werchan M, Zuberbier T. The distribution of pollen and particulate matter in an urban agglomeration using the city of Berlin as an example. Allergo J. 2013; 22: 471–475.

[49] Hamid N, Ali SM, Talib F, Sadiq I, Ghufran MA. Spatial and temporal variations of pollen concentrations in Islamabad (Pakistan): effect of meteorological parameters and impact on human health. Grana. 2015; 54(1): 53–67.

[50] Peel RG, Kennedy R, Smith M, Hertel O. Do urban canyons influence street level grass pollen concentrations? Int J Biometeorol. 2014; 58(6): 1317–1325. doi: 10.1007/s00484-013-0728-x 24037300 10.1007/s00484-013-0728-x

[51] Skjøth C, Ørby PV, Becker T, Geels C, Schlünssen V, Sigsgaard T, et al Identifying urban sources as cause of elevated grass pollen concentrations using GIS and remote sensing. Biogeosciences. 2013; 10: 541–554.

[52] Simoleit A, Gauger U, Mücke HG, Werchan M, Obstova′ B, Zuberbier T, et al Intradiurnal patterns of allergenic airborne pollen near a city motorway in Berlin, Germany. Aerobiologia. 2016; 32(2): 199–209.

[53] Munoz Rodriguez AF, Palacios I, Molina R. Influence of meteorological parameters in hourly patterns of grass (Poaceae) pollen concentrations. Ann Agric Environ Med. 2010; 17(1): 87–100. 20684485

[54] IPCC: Climate Change 2013: The Physical Science Basis. Contribution of Working Group I to the Fifth Assessment Report of the Intergovernmental Panel on Climate Change. In: Stocker TF, Qin D, Plattner G-K, Tignor M, Allen SK, Boschung J, et al. editors. Cambridge and New York: Cambridge University Press; 2013.

